# Supplementary material for: Single-molecule functional anatomy of endogenous HER2-HER3 heterodimers
Source: eLife. 2020 Apr 8;9:e53934. doi: 10.7554/eLife.53934 (PMC7176432; doi:10.7554/eLife.53934)
Supplement: Supplementary file 1. [file elife-53934-supp1.docx]

| **Key Resources Table** | | | | |
| --- | --- | --- | --- | --- |
| **Reagent type (species) or resource** | **Designation** | **Source or reference** | **Identifiers** | **Additional information** |
| antibody | Goat Anti-GFP polyclonal (biotin-conjugated) | Abcam | Cat#ab6658; RRID:AB_305631 | 1/1000 for use |
| antibody | Mouse anti-human HER2 (biotin-conjugated, clone:2G11) | Thermo Fisher Scientific | Cat#BMS120BT; RRID:AB_10598667 | 1/100 for use |
| antibody | Mouse anti-human HER3 (biotin-conjugated, clone:66201) | R&D Systems | Cat#BAM348; RRID:AB_356892 | 1/100 for use |
| antibody | Rabbit anti-human HER2 (clone:K.929.9) | Thermo Fisher Scientific | Cat#MA5-15050; RRID:AB_10989303 | 1/100 for use |
| antibody | Rabbit anti-human HER3 (clone:E186) | Abcam | Cat#ab32121; RRID:AB_11160022 | 1/100 for use |
| antibody | Rabbit anti-human HER2 pY1139 (clone:EP1046Y) | Abcam | Cat#ab53290; RRID:AB_869096 | 1/100 for use |
| antibody | Rabbit anti-human HER2 pY1196 (clone:D66B7) | Cell Signaling Technology | Cat#6942S; RRID:AB_10860762 | 1/100 for use |
| antibody | Rabbit anti-human HER2 pY1221/22 (clone:6B12) | Cell Signaling Technology | Cat#2243S; RRID:AB_490899 | 1/100 for use |
| antibody | Rabbit anti-human HER2 pY1248 Polyclonal | Cell Signaling Technology | Cat#2247; RRID:AB_331725 | 1/100 for use |
| antibody | Rabbit anti-human HER3 pY1197 (clone:EPR5806) | Abcam | Cat#ab133444; RRID:- | 1/100 for use |
| antibody | Rabbit anti-human HER3 pY1222 (clone:EPR5807) | Abcam | Cat#ab133445; RRID:- | 1/100 for use |
| antibody | Rabbit anti-human HER3 pY1276 (clone:EPR5808(2)) | Abcam | Cat#ab134900; RRID:- | 1/100 for use |
| antibody | Rabbit anti-human HER3 pY1289 (clone:21D3) | Cell Signaling Technology | Cat#4791S; RRID:AB_2099709 | 1/100 for use |
| antibody | Rabbit anti-human HER3 pY1328 (clone:E1J1T) | Cell Signaling Technology | Cat#14525S; RRID:- | 1/100 for use |
| antibody | Goat anti-Rabbit IgG Fc Specific polyclonal(Cy3 conjugated) | Jackson ImmunoResearch Labs | Cat#111-165-046; RRID:AB_2338004 | At 10nM |
| antibody | Donkey anti-Rabbit IgG (H+L) polyclonal (Cy3 conjugated) | Jackson ImmunoResearch Labs | Cat#711-165-152;  RRID:AB_2307443 | At 10nM |
| antibody | Donkey anti-Mouse IgG (H+L) polyclonal (Cy3 conjugated) | Jackson ImmunoResearch Labs | Cat#715-165-150;  RRID:AB_2340813 | At 10 nM |
| Chemical compound, drug | ATP disodium trihydrate | VWR | Cat#97061-224 |  |
| Chemical compound, drug | (±)-6-hydroxy-2,5,7,8-tetramethylchromane-2-carboxylic acid | Sigma Aldrich | Cat#238813 |  |
| Chemical compound, drug | biotin - m - Poly Ethylene Glycol 5000 - SVA | LaySan Bio | Cat#BIO-PEG-SVA-5K |  |
| Chemical compound, drug | CHAPSO | Sigma Aldrich | Cat#C3649 |  |
| Chemical compound, drug | Coenzyme A trilithium salt | Sigma Aldrich | Cat#C3019 |  |
| Chemical compound, drug | Coverslip | VWR | Cat#48393251 |  |
| Chemical compound, drug | Cyanine3 maleimide | Lumiprobe | Cat#21080 |  |
| Chemical compound, drug | Cyanine5 maleimide | Lumiprobe | Cat#23080 |  |
| Chemical compound, drug | DDM | Anatrace | Cat#D310 |  |
| Chemical compound, drug | Digitonin | Sigma Aldrich | Cat#D141 |  |
| Chemical compound, drug | DMEM | Gibco | Cat#11995-073 |  |
| Chemical compound, drug | DMSO | Sigma Aldrich | Cat#276855 |  |
| Chemical compound, drug | EDTA | Sigma Aldrich | Cat#EDS |  |
| Chemical compound, drug | Fetal Bovine Serum | Life Technologies | Cat#26140-079 |  |
| Chemical compound, drug | GDN | Anatrace | Cat#GDN101 |  |
| Chemical compound, drug | Gentamicin | Life Technologies | Cat#15710-063 |  |
| Chemical compound, drug | Glycerol | JUNSEI | Cat#27210S0350 |  |
| Chemical compound, drug | HEPES | LPS Solution | Cat#HEPE500 |  |
| Chemical compound, drug | Magnesium chloride hexahydrate | Sigma Aldrich | Cat#M2670 |  |
| Chemical compound, drug | m-Poly Ethylene Glycol 5000 - SVA | LaySan Bio | Cat#mPEG-SVA-5K |  |
| Chemical compound, drug | N - [3 - (Trimethoxysilyl))propyl] ethlenediamine | Sigma Aldrich | Cat#104884 |  |
| Chemical compound, drug | OG | Glycon | Cat#D97001 |  |
| Chemical compound, drug | Phosphatase Inhibitor Cocktail 2 | Sigma Aldrich | Cat#P5726 |  |
| Chemical compound, drug | Protease Inhibitor Cocktail | Sigma Aldrich | Cat#P8340 |  |
| Chemical compound, drug | Protocatechuate 3,4-dioxygenase (PCD) | Sigma Aldrich | Cat#P8279 |  |
| Chemical compound, drug | Protocatechuic acid (PCA) | Sigma Aldrich | Cat#03930590 |  |
| Chemical compound, drug | Quartz Slide | Finkenbeiner | Cat#1"X3"X1mmTHICK |  |
| Chemical compound, drug | RPMI1640 | Life Technologies | Cat#22400-105 |  |
| Chemical compound, drug | Sodium chloride | Sigma Aldrich | Cat#746398 |  |
| Chemical compound, drug | Triton - X - 100 | Sigma Aldrich | Cat#X100 |  |
| Chemical compound, drug | TrypLE Express Enzyme | Thermo Fisher Scientific | Cat#12605010 |  |
| Peptide, recombinant protein | Biotin-(miniPEG2)*2-HER2 pY1139 peptide | Peptron | Custom odered | PQPEpYVNQPDVRd |
| Peptide, recombinant protein | 4'-phosphopantetheinyl transferase (Sfp) | New England Biolabs | Cat#P9302 |  |
| Peptide, recombinant protein | Bovine Serum Albumin | Sigma Aldrich | Cat#A2153 |  |
| Peptide, recombinant protein | NeutrAvidin | LifeTechnologies | Cat#A2666 |  |
| Peptide, recombinant protein | Recombinant human Neuregulin 1 beta 1 (NRG1-β1) | ProspecBio | Cat#Cyt733 |  |
| Peptide, recombinant protein | Recombinant human PTPN1 | ProspecBio | Cat#Pka219 |  |
| Commercial assay, kit | DC Protein assay (Reagent A, B, S) | Bio-Rad | Cat#5000113, Cat#5000114, Cat#5000115 |  |
| Commercial assay, kit | Zeba^TM^ Spin Desalting Columns | Thermo Scientific | Cat#87766 |  |
| Commercial assay, kit | Mycoplasma PCR detection kit | Lilif | Cat#25237 |  |
| Cell line | HEK - 293T | Donated from W.D. Heo | RRID:CVCL_0063 |  |
| Cell line | SK - BR - 3 | Korean Cell Line Bank and ATCC | RRID:CVCL_0033 |  |
| Sequence-based reagent | Primer: HER2linkerybbRJM_F | Macrogen, Inc. | N/A | ATTCTCTTGAATTTATTGCTAGTAAGCTTGCGGGTGGAGGCGAGCTGGTGGAGCCGCTGACACCT |
| Sequence-based reagent | Primer: HER2linkerybbRJM_R | Macrogen, Inc. | N/A | ACTAGCAATAAATTCAAGAGAATCCAACACGGTACCTCCGCCCGTTTCCTGCAGCAGTCTCCGCA |
| Sequence-based reagent | Primer: HER3linkerybbRJM_F | Macrogen, Inc. | N/A | GATTCTCTTGAATTTATTGCTAGTAAGCTTGCGGGTGGAGGCGAGAGCATAGAGCCTCTGGACCC |
| Sequence-based reagent | Primer: HER3linkerybbRJM_R | Macrogen, Inc. | N/A | CTTACTAGCAATAAATTCAAGAGAATCCAACACGGTACCTCCGCCACCCCGTTCCAAGTATCGCC |
| Software, algorithms | HMM algorithm for single-molecule fluorescence signal analysis | (Lee, 2009) | N/A |  |
